# Structural Elucidation of Heteropolysaccharides from the Peach-Shaped *Dictyophora indusiata* and Its Anti-Inflammatory Activity

**DOI:** 10.3390/foods14091536

**Published:** 2025-04-27

**Authors:** Ying He, Hao Yang, Yaxin Liu, Yanting Sun, Zeguo Feng, Xueying Zheng, Fei Wang, Lei Ma, Jianbao Zhang, Dan Xu, Hui Guo, Liguo Qin, Yali Zhang

**Affiliations:** 1 Center for Mitochondrial Biology and Medicine, Key Laboratory of Biomedical Information Engineering of Ministry of Education, School of Life Science and Technology, Xi’an Jiaotong University, Xi’an 710049, China; heying2018@stu.xjtu.edu.cn (Y.H.);; 2 Key Laboratory of Education Ministry for Modern Design and Rotor-Bearing System, Institute of Design Science and Basic Components, School of Mechanical Engineering, Xi’an Jiaotong University, Xi’an 710049, China; yhz19950607@stu.xjtu.edu.cn; 3 Department of Endocrinology, First Affiliated Hospital of Medical College, Xi’an Jiaotong University, Xi’an 710061, China

**Keywords:** peach-shaped phase of *Dictyophora indusiata*, 1,3-β-glucan, stability, human U937 cells, anti-inflammatory

## Abstract

*Dictyophora indusiata* is commonly utilized as a functional food in China and other Asian countries. The peach-shaped phase of this fungus is nutritionally and taste-wise similar to its mature fruiting bodies. However, there is limited research on the polysaccharides found in the peach-shaped *D. indusiata*. A heteropolysaccharide was extracted from the volva of peach-shaped *D. indusiata* (DIVP). Analyses using high-performance gel permeation chromatography, methylation and NMR revealed that DIVP comprises glucose, glucuronic acid, galactose, and mannose. Its structure features a backbone that consists of →3)-β-D-Glc*p*-(1→ units with branches at →4)-β-D-Glc*p*-(1→, →6)-α-D-Gal*p*-(1→ and terminal α-Man*p*-(1→ residues. Physicochemical assessments including X-ray diffraction, thermal, zeta potential and viscosity characterization indicated that DIVP is a semi-crystalline polymer exhibiting excellent physical and thermal stability. Cytokine antibody array and proteome profiler human phosphokinase analyses demonstrated that DIVP downregulates the expression levels of cytokines and alters the phosphorylation status of 16 proteins in human U937 macrophages induced by lipopolysaccharides, indicating its anti-inflammatory activity. These findings suggest that the polysaccharide from the volva of peach-shaped *D. indusiata* is primarily composed of β-1,3-glucan, which exhibits stable physicochemical properties and anti-inflammatory activity, providing a foundation for its potential use as an anti-inflammatory agent or functional food.

## 1. Introduction

Bioactive polysaccharides are abundant in microorganisms, plants, and animals. They are widely recognized for numerous health benefits, including anti-inflammatory, immunomodulatory, antioxidant, anti-diabetic, and probiotic-like effects [1,2,3]. *Dictyophora indusiata* (*D. indusiata*), a member of the Phalaceae family within the order Phalales, is highly esteemed as one of the most valuable edible mushrooms due to its exquisite appearance and diverse range of bioactive compounds, the polysaccharides derived from which have been shown to play a significant role in various functions, including antiinflammatory, antioxidant, and antibacterial activities [4].

The structural and functional properties of polysaccharides can vary significantly based on the biology classification, the growth stage, and the extraction parts of the raw materials [5]. Sun et al. discovered that polysaccharides extracted from the pileus of mature *Dictyophora rubrovoltata* are a promising source rich in potent anti-glycation and antioxidant compounds [6]. Several polysaccharides isolated from the fruiting bodies of mature *D. indusiata* feature a backbone predominantly composed of 1,3-β-D-glucan, with side chains consisting of either 1,4-β and/or 1,6-β-D-glucan [7,8], or comprising mannose and galactose side chains [9]. These polysaccharides have been shown to enhance nitric oxide (NO) production while increasing levels of the cytokines including TNF-α, IL-1, IL-6, and IL-12 in RAW264.7 cells [7] through specific binding to complex receptor 3 [9]. Furthermore, they also exhibit anti-inflammatory effects by inhibiting the TLR4/NF-κB signaling pathway and preventing the activation of the NLRP3 inflammasome [8].

**Figure 1:**
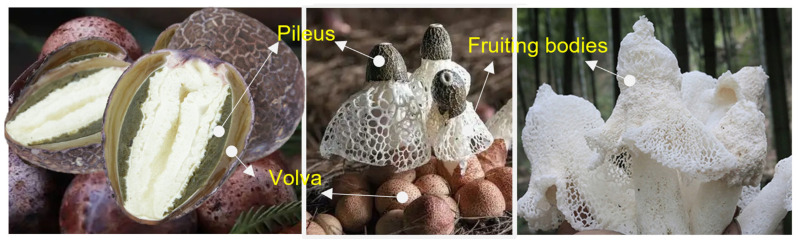
Peach-shaped buds (**left**), mycelia (**middle**), and fruiting bodies (**right**) of *D. indusiata*.

Two notable features emerge from the existing literature. First, all studied polysaccharides were exclusively derived from the fruiting bodies of mature *D. indusiate*. Secondly, the cellular models employed in the anti-inflammatory research predominantly utilize mouse RAW 264.7 macrophages. The developmental stages of *D. indusiata* include the primitive stage, spherical stage, peach-shaped stage, and mature stage [3]. At the end of the peach-shaped stage, the outermost layer surrounding the *D. indusiata* buds develops into its volva and pileus covered with spores, which may contain essential nutrients and health-promoting compounds (Figure 1), such as dendronobilin and indole acetic acid [10]. However, this part is often discarded due to a perceived lack of utility. The quality of mature fruiting bodies deteriorates rapidly due to aging and self-decomposition [5,11]. The peach-shaped *D. indusiata*, commonly known as bamboo fungus egg, is recognized for its superior nutritional and medicinal value. Currently, a diverse array of commercially available products derived from it exists [12,13]. To our knowledge, there is limited information regarding the structural characteristics and bioactivity associated with the polysaccharides found in the volva of peach-shaped *D. indusiata*.

Previous studies have shown that polysaccharides derived from *D. indusiata* exhibit immunomodulatory, antioxidant, and anti-inflammatory activities in rodent models of non-communicable diseases (NCDs) [3,14,15,16], where macrophage activation is associated with pathophysiological processes. However, the direct effects of *D. indusiata* polysaccharides on human macrophage activation have not been investigated. U937 cells, a type of human histiocytic lymphoma cell, closely resemble human cellular composition, structure, and antigen response. Therefore, they are widely utilized as a model for studying monocyte–macrophage differentiation and function [17]. In this study, we isolated a novel heteropolysaccharide from the volva of *D. indusiata* during its peach-shaped stage, designated as DIVP, differing from previous studies that focused on the matured *D. indusiata*. Initially, we evaluated the anti-inflammatory properties of the heteropolysaccharide extracted from the volva, contrasting with earlier studies that primarily examined the fruit body. Additionally, the cell model employed for assessing the anti-inflammatory effects of DIVP was human U937 cells induced by lipopolysaccharide (LPS), which differs from the mouse RAW264.7 model used in prior research, making our findings more relevant to human physiology. Moreover, our study utilized advanced techniques such as inflammatory cytokine dot blot arrays and phosphokinase antibody arrays, which identified more key anti-inflammatory factors and proteins. Overall, this research provides valuable insights into the properties of DIVP and its potential applications in the food and pharmaceutical industries.

## 2. Materials and Methods

### 2.1. Preparation of DIVP

*D. indusiata* fungus buds were purchased from Kunming, Yunnan, China in June 2021 and identified by Prof. Yali Zhang from Xi’an Jiao tong University. The volva of peach-shaped *D. indusiata* was immersed in water at 40 °C, achieving a final ratio of 1:30. Following overnight incubation, a complex enzyme solution including 1.5% cellulase (15 U/mg), 2% neutral protease (50 U/mg) and 1.5% pectinase (20 U/mg) was applied for enzymatic hydrolysis for 1 h, which was followed by digestion at 60 °C for 3 h. The resulting mixture was then filtered and concentrated to achieve a solid-to-liquid ratio of 1:3. Ethanol was added in a threefold volume, and the solution was allowed to stand overnight. The precipitate obtained was dissolved, and proteins were removed using Sevag reagent solution at a 1:5 volume ratio. The crude polysaccharide was then collected and precipitated using 80% ethanol (*v*/*v*), which was followed by purification on a DEAE-cellulose column (Sigma, St. Louis, MO, USA) and a Sephadex G-75 column (Sigma, St. Louis, MO, USA). The eluent containing the polysaccharides underwent lyophilization to yield DIVP [18].

### 2.2. Characteristics of DIVP

#### 2.2.1. Molecular Weight (Mw) and Monosaccharide Composition Analysis

The molecular weight and purity of the polysaccharides were determined using high-performance gel permeation chromatography (HPGPC) according to the previous method with little modifications: DIVP (10 L) was injected and run with deionized water at 0.6 mL/min as the mobile phase. The standard curve was established by using T-series Dextran as the standards (T-10, T-40, T-70, T-500 and T-2000). The molecular weight of each composition was calculated by contrast with the retention time of the monosaccharide reference standard [19].

PMP (3-Methyl-1-phenyl-2-pyrazoline-5-one)-labeled monosaccharides were analyzed using an ICS5000 Ion Chromatography system (Thermo Fisher Scientific Co., Ltd., Waltham, MA, USA) according to a reported method with slight modifications [20]. The analytical column was the Dionex CarbopacTMPA20 (3 mm × 150 mm, Thermo Fisher Scientific Co., Ltd., Waltham, MA, USA). The mobile phase consisted of (A) H_2_O; (B) 15 mM NaOH; and (C) 15 mM NaOH with 100 mM NaOAc with a flow rate of 0.3 mL/min and an injection volume of 5 µL. The temperature was maintained at 30 °C while employing an electrochemical detector for analysis. Monosaccharide standards, including fructose, glucose, galactose, rhamnose, mannose, xylose, arabinose, fucose, ribose, glucuronic acid, galacturonic acid, and mannuronic acid, were prepared using a standardized procedure to quantify the monosaccharide content based on their corresponding peak areas and response factors.

#### 2.2.2. Methylation Analysis

Methylation analysis was conducted using a GC-MS 6890-5973 (Agilent Technologies Co., Ltd., Palo Alto, CA, USA) equipped with an RXI-5sil MS capillary column (30 m × 0.25 mm × 0.25 µm) according to the previous method with little modification [21]. The temperature program began with at an initial column temperature of 120 °C, which was then increased to 250 °C at a rate of 3 °C/min. This final temperature was maintained for 5 min. Both the injection port and detector temperatures were set at 250 °C, while the column flow rate was maintained at 1.0 mL/min.

#### 2.2.3. Nuclear Magnetic Resonance Spectroscopy (NMR) Analysis

First, 50 mg of DIVP was dissolved in 0.55 mL of D_2_O for NMR analysis. ^1^H NMR, ^13^C NMR, ^1^H-^1^H HSQC, HMBC, COSY, and NOESY spectra were recorded using a Bruker 600 MHz NMR spectrometer (Bruker Corp., Fallanden, Switzerland) with acetic acid serving as the internal standard at 25 °C [22].

#### 2.2.4. Observation of Morphology and Microstructure

Following lyophilization and gold-sputtering procedures, the morphology and microstructure of DIVP were examined using a scanning electron microscope (SEM, MAIA3 LMH, Brno, Czech Republic) operating at a voltage of 15 kV [23]. Additionally, the surface characteristics were investigated using an atomic force microscope (AFM, SPM-9700HT, Kyoto, Japan).

#### 2.2.5. X-Ray Diffraction (XRD) Analysis

The crystalline structure was determined using a Bruker X-ray diffraction analyzer (D8, Bruker, Berlin, Germany) under specific conditions. A copper target was employed as the irradiation source with tube voltage set at 40 kV and the tube current maintained at 30 mA. The scanning speed was established at 15 °C/min, and the scanning range was configured from 2θ = 5–60° [24].

#### 2.2.6. Thermal Analysis

The thermal stability properties of DIVP were evaluated using a NETZSCH thermos gravimetric analyzer (TG 209 F3 Tarsus^®^, NETZSCH, Hanau, Germany). The experimental conditions included a temperature range of 30 to 600 °C with a heating rate (β) of 5 °C/min in a nitrogen atmosphere and a gas flow rate of 250.0 mL/min [25].

#### 2.2.7. Zeta Potential Analysis

The zeta potential was analyzed using a nanoparticle measurement instrument (Zetasizer Nano ZS90, Malvern Panalytical, Malvern, United Kingdom). A concentration of DIVP at 0.1 mg/mL was adjusted to pH values ranging from 4.0 to 10.0 using 1 M HCl and 1 M NaOH [26].

#### 2.2.8. Viscosity Characterization Analysis

The viscosity was measured using a rotational rheometer (Kinexus Lab^+^, NETZSCH, Hanau, Germany) according to the methods with little modifications [27]. The diameter of the parallel plate holder was set to 40 mm with an inter-plate gap of 0.5 mm. The shear rate of the DIVP solution was tested over a range of 0.1 to 100 s^−1^ at a temperature of 25 °C.

### 2.3. Anti-Inflammatory Activity of DIVP in Human U937 Cells

#### 2.3.1. Cell Culture and Differentiation Induction

U937 cells (ATCC CRL-1593) were cultured in a 35 mm cell culture plate using RPMI medium supplemented with 10% fetal bovine serum (FBS) and incubated at 37 °C in an atmosphere containing 5% CO_2_. To induce differentiation into a macrophage-like phenotype, U937 cells were treated with PMA at a concentration of 0.2 µg/mL for 3 days, as described by Córdova-Dávalos et al. [28].

#### 2.3.2. Cell Viability Analysis

Cells were seeded in a 96-well plate at a density of 1 × 10^5^ cells/mL and exposed to various concentrations of DIVP (0.2, 0.4, 0.6, 0.8, 1, 1.5, or 2 mg/mL). After incubating for 48 h, 20 µL of MTT solution (5 mg/mL) was added to each well, which was followed by additional incubation for 4 h. Absorbance was measured at 490 nm after the addition of 200 µL of DMSO [29].

#### 2.3.3. Assay of Phagocytic Activity

The cells were plated at a density of 3 × 10^3^ per well in a 96-well plate and incubated at 37 °C with 5% CO_2_. The treatment groups received 100 µL of DIVP or 10 ng/mL LPS (Sigma-Aldrich Corp, St. Louis, MO, USA) for a duration of 24 h. Subsequently, the pretreated cells were incubated with 100 μL of a neutral red solution (0.01%) (Beyotime Biotechnology, Shanghai, China) for 2 h at 37 °C. Absorbance was measured at 540 nm [30].

#### 2.3.4. Quantitative Reverse Transcription Polymerase Chain Reaction (qRT-PCR)

U937 cells were treated with either 0.2 mg/mL or 2 mg/mL of DIVP, along with 10 ng/mL of LPS, for an additional 24 h. Total RNA was extracted using TRIzol™ reagent (Sigma Chemical Co., St. Louis, MO, USA), and cDNA synthesis was performed using Evo M-MLV RT Premix (AG Company, Beijing, China). Quantitative analysis of cDNA production for TNF-α, IL-1β, IL-6, and IL-8, which are indicators for the level of inflammation, was conducted using quantitative real-time PCR with β-actin serving as an internal reference gene throughout the process. The primer sequences used are shown in Table 1.

#### 2.3.5. Dot Blot Array for the Detection of Inflammatory Cytokines

The levels of soluble cytokines secreted by LPS-stimulated U937 cells, both in the presence and absence of DIVP, were analyzed using the Human Cytokine Array Kit panel A (ARY005B, R&D Systems, Minneapolis, MN, USA) following the manufacturer’s instructions. Pixel density quantification for each spot was performed using NIH ImageJ version 2.0 software. Positive control spots on each membrane served as references to normalize results across different membranes, enabling us to average intensity measurements over duplicate spots.

#### 2.3.6. Phosphokinase Antibody Array

Protein phosphorylation was evaluated using phosphokinase antibody array assays (ARY003C, R&D Systems, Minneapolis, MN, USA) according to the manufacturer’s instructions. Briefly, protein lysates (800 μg) from U937 cells were hybridized and incubated overnight at 4 °C. Membranes was washed with 1 × Wash Buffer. Subsequently, the membranes were incubated with Streptavidin–HRP for 30 min at room temperature, and then chemiluminescence detection was conducted.

### 2.4. Statistical Analysis

Data were obtained from at least three independent experiments and expressed as mean ± standard deviation (S.D.). The significance of differences between experimental groups was assessed using a one-way analysis of variance (ANOVA) with the SPSS 16.0 software package (IBM, Armonk, NY, USA). The data were plotted with Graphpad prism Version 6.01 (GraphPad Software Inc., Boston, MA, USA) and the SPSS 16.0 software package (IBM, USA). A *p*-value of less than 0.05 was considered statistically significant.

## 3. Results and Discussion

### 3.1. Structural Characteristics of DIVP

**Figure 2:**
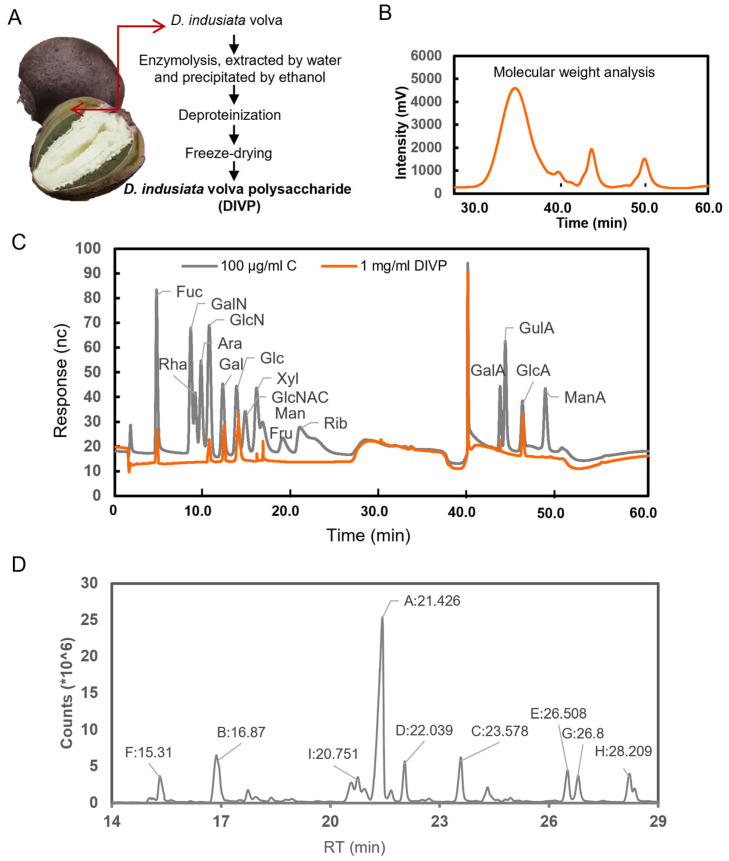
General structural characteristics of DIVP. (**A**) Schematic illustration of the preparation of DIVP. (**B**) Molecular weights. (**C**) Monosaccharide components, profiles of standard monosaccharides were shown in gray as reference, and DIVP in orange. (**D**) Methylated analysis.

Crude polysaccharide was extracted from the volva of peach-shaped *D. indusiata* through a series of steps, including water extraction, ethanol precipitation, deproteinization, decolorization, and lyophilization (Figure 2A). The total polysaccharide content of DIVP is determined to be 96.56%. HPGPC analysis displays a single symmetrical peak, indicating a homogeneous polysaccharide (Figure 2B). The Mw for DIVP is found to be 96.6 kDa, with a molecular weight distribution (Mw/Mn = 1.56), suggesting a satisfactory degree of polymerization and high purity. The analysis of monosaccharide standards indicates that DIVP contains glucose, glucuronic acid, galactose, mannose, xylose, galacturonic acid, fucose, and glucosamine hydrochloride in a molar ratio of 0.535:0.308:0.06:0.047:0.02:0.013:0.009:0.008 (Figure 2C).

The total ion chromatograms (TICs) of PMAAs identified in DIVP are presented in Figure 2D, which are accompanied by mass spectrometry (MS) fragments. Based on databases from GC-EIMS for PMAAs provided by the Complex Carbohydrate Research Center at the University of Georgia, along with recent studies [31], Table 2 lists the PMAA derivatives including 2,4,6-Me3-Glc*p*, 2,3,4,6-Me4-Glc*p*, 2,3,4-Me3-Gal*p*, 2,3,6-Me3-Glc*p*, 2,3-Me2-Glc*p*, 2,3,4,6-Me4-Man*p*, 2,4-Me2-Glc*p*, 2,4-Me2-Gal*p*, and 3,4,6-Me3-Glc*p* with a molar ratio of 45.6:11.2:7.0:5.6: 5.4:5.3:5.1: 5.1: 4.1. Notably, the predominant derivative is identified as 2,4,6-tri-Me-glucitol (45.6%), suggesting that units linked via 1,3-linked glucopyranosyl residues form a primary structural component within DIVP.

**Figure 3:**
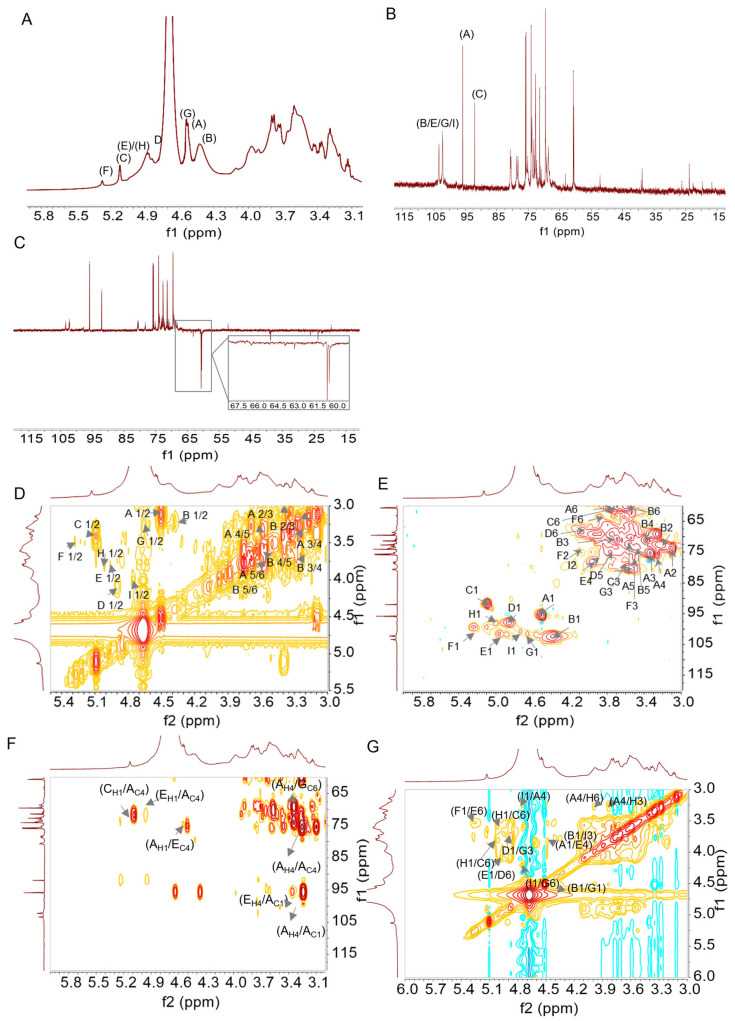
NMR spectra of DIVP. (**A**), ^1^HNMR; (**B**), ^13^C NMR; (**C**), DEPT135; (**D**), H-HCOSY; (**E**), HSQC; (**F**), HMBC; and (**G**), NOSY. ((A), (B), (C), (D), (E), (F), (G), (H), and (I) represent the following residues: →3)-Glc*p*-(1→, Glc*p*-(1→, →6)-Gal*p*-(1→, →4)-Glc*p*-(1→, →4,6)-Glc*p*-(1→, Man*p*(1→,→3,6)-Glc*p*-(1→, →3,6)-Gal*p*-(1→, and →2)-Glc*p*-(1→.).

To further elucidate and validate these structural features, NMR analysis was performed. The C/H chemical shifts corresponding to the major glycosidic linkages in DIVP are assigned based on literature and methylation analysis [32], as detailed in Figure 3, and summarized in Table 3.

According to the ^1^H NMR spectrum (Figure 3A) and the cross-peaks observed in the H-H COSY NMR spectrum (Figure 3D), the anomeric proton region (δ 5.5–4.3 ppm) exhibit nine primary signals at δ 5.25, 5.09, 4.99, 4.97, 4.86, 4.73, 4.66, 4.51, and 4.39 ppm [31]. The corresponding ^13^C NMR spectrum (Figure 3B) contains five anomeric carbon signals at δ 103.38, δ 102.24, δ 97.87, δ 95.85, and δ 92.03 ppm. Notably, the peaks at δ 103.38 ppm, δ 102.24 ppm, and δ 95.85 ppm exhibit a split pattern, suggesting their likely origin from the same type of sugar existing in slightly different structural environments [33]. The HSQC spectrum (Figure 3E) presents nine distinct ^1^H/^13^C cross-peaks within the anomeric region, including δ 4.39/102.24 ppm, δ 4.51/95.84 ppm, δ 4.66/101.58 ppm, δ 4.73/100.67 ppm, δ 4.86/97.87 ppm, δ 4.97/101.58 ppm, δ 4.99/98.05 ppm, δ 5.09/91.76 ppm, and δ 5.24/99.32 ppm, respectively. These findings indicate the presence of nine types of glycosidic residues, which are designated as B, A, G, I, D, E, H, C, and F residues [34,35]. Furthermore, the absence of any signals in the range of δ 82–88 ppm provides evidence that all sugar residues are present in pyranose form [36]. A weak characteristic signal of uronic acid (~δ 176 ppm) is observed, indicating that DIVP is an acidic polysaccharide [37]. The DEPT-135 spectrum reveals positive CH_3_ and CH signals, negative CH_2_ signals, and inverted peaks assigned to C6 of residues C, D, E, G, and H at different chemical shifts (Figure 3C). Resonance signals for both the anomeric proton at δ 4.4–5.3 ppm and the anomeric carbon at δ 90–110 ppm confirm the presence of both the α-configuration (δ 4.9–5.3 ppm) and the β-configuration (δ 4.4–4.9 ppm) in DIVP [38]. The chemical shifts observed at δ 4.51/95.84, 4.39/102.24, 4.89/97.87, 4.97/101.5, 4.66/101.58, and 4.73/100.67 ppm in the HSQC spectrum (Figure 3E) are assigned to H1 and C1, indicating the β-configuration of sugar residues A, B, D, E, G, and I [39].

The H-H COSY spectrum (Figure 3D) reveals that the chemical shifts at δ 3.12, 3.32, 3.26, 3.56, and 3.75 ppm corresponded to H2, H3, H4, H5, and H6 of residue A, respectively. Meanwhile, the shifts corresponding to C2, C3, C4, C5, and C6 of residue A appear at δ 73.77, 75.23, 77.34, 72.50, and 60.44 ppm in the HSQC spectrum (Figure 3E). Additionally, the H-H COSY spectrum indicates that the chemical shifts observed at δ 3.22, 3.69, 3.39, 3.48, and 3.60 ppm are assigned to H2, H3, H4, H5, and H6 of residue B, respectively. The chemical shifts corresponding to C2, C3, C4, C5, and C6 of residue B appear at δ 72.61, 70.87, 71.16, 73.52, and 60.51 ppm in the HSQC spectrum. The H-H COSY spectrum also shows that the chemical shifts for H2, H3, H4, H5, and H6 of residue D are δ 3.54, 3.70, 3.31, 3.77, and 4.09 ppm, respectively, while the chemical shifts of C2, C3, C4, C5, and C6 of residue D appear at δ 70.43, 73.52, 71.16, 74.79, and 68.43 ppm in the same HSQC spectrum. By comparing the experimental proton and carbon resonances with literature data [40], residues A, B, and D are identified as →3)-β-D-Glc*p*-(1→, β-D-Glc*p*-(1→, and →4)-β-D-Glc*p*-(1→, respectively. The complete ^1^H and ^13^C chemical shifts for residues E, G, and I are consistent with previous reports [41], confirming that residues E, G, and I are linked as →4,6)-β-D-Glc*p*-(1→, →3,6)-β-D-Glc*p*-(1→, and →2)-β-D-Glc*p*-(1→, respectively.

Moreover, the signals for residues C (δ 5.09/91.76 ppm) and H (δ 4.99/98.05 ppm) correspond to two distinct galactose residues. The H-H COSY spectrum reveals H1/H2 coupling for residues C and H at δ 5.09/3.42 ppm and δ 4.99/3.79 ppm, respectively. The HSQC spectrum demonstrates H2/C2 shifts at δ 3.42/72.98 ppm (residue C) and δ 3.79/72.79 ppm (residue H), suggesting that residue C is →6)-α-D-Gal*p*-(1→. Concurrently, the downfield chemical shifts of C-3 and C-6 indicate that residue H corresponds to →3,6)-α-D-Gal*p*-(1→ [42,43,44].

Furthermore, the anomeric signals of residue F, located at 5.24/99.32 ppm, exhibit H2-H6 signals at δ 4.10, 3.52, 3.49, 3.74, and 3.86 ppm, as indicated by the H-H COSY spectrum. Additionally, the C2-C6 signals for this residue are observed at δ 74.61, 76.24, 68.73, 76.35, and 63.89 ppm, as evidenced by the HSQC spectrum. The identity of residue F is confirmed through a comparison with literature data, which indicates that it is terminal α-D-Manp [45,46,47].

Based on the chemical shifts of sugar residues in the ^1^H and ^13^C NMR spectra, combined with the HMBC spectrum (Figure 3F) and the NOESY spectrum (Figure 3G), the structure and linkage types of DIVP are elucidated. The cross-peak signals observed at δ 4.51/77.34 ppm for sugar residues A-H1 and A-C4, along with the cross-peak signals at δ 95.84/3.26 ppm for sugar residues A-C1 and A-H4, are identified in the HMBC spectrum. This indicates that the DIVP backbone contains a →3)-β-D-Glc*p*-(1→3)-β-D-Glc*p*-(1→ glycosidic linkage. Furthermore, the observation of cross-peaks A-H1/E-C4 at δ 4.51/78.06 ppm and E-H4/A-C1 at δ 3.75/95.84 ppm suggests the possible presence of a →3)-β-D-Glc*p*-(1→4,6)-β-D-Glc*p*-(1→ linkage. The H1 and C1 of →6)-α-D-Gal*p*-(1→ exhibit correlations with the C4 and H4 of →3)-β-D-Glc*p*-(1→, indicating the existence of a glycosidic linkage between →6)-α-D-Gal*p*-(1→ and →3)-β-D-Glc*p*-(1→. Additionally, the correlation peak observed between the H1 of α-D-Man*p*-(1→ and the H6 of →4,6)-β-D-Glc*p*-(1→ further suggests the presence of an α-D-Man*p*-(1→4,6)-β-D-Glc*p*-(1→ linkage. Moreover, related peaks observed between H3/C3 and H6/C6 from different sugar residues lead to the conclusion that various other glycosidic linkages are present within DIVP.

In conclusion, the analysis that combined methylation data with NMR interpretation indicates that the main chain consists of repeated units structured as →3-β-D-Glc*p*-1→. The branched chains exhibit linkages characterized as follows: →4,6)-β-D-Glc*p*-(1→6)-β-Glc*p*-1→4)-β-D-Glc*p*-(1→ and →3,6)-α-D-Gal*p*-(1→6)-α-D-Gal*p*-(1→. Additionally, terminal α-Man*p*-(1→ residues are identified within these side chains. This structural composition closely resembles the polysaccharide found in the fruiting bodies of mature *D. indusiata* more than those present in the discarded volva tissues [15,48]. This observation suggests that during the maturation of *D. indusiata*, there may be a transformation from 1,3-β-glucan into fibrils primarily composed of 1,4-β-glucan, which consequently leads to a loss of specific characteristics associated with 1,3-β-glucan, and during the maturation process of *D. indusiata*, the structure of the volva polysaccharides has changed, which may cause functional changes. Further research on this modification process is needed to provide evidence for the study of the structure–activity relationship.

### 3.2. Physiochemical Characteristics of DIVP

**Figure 4:**
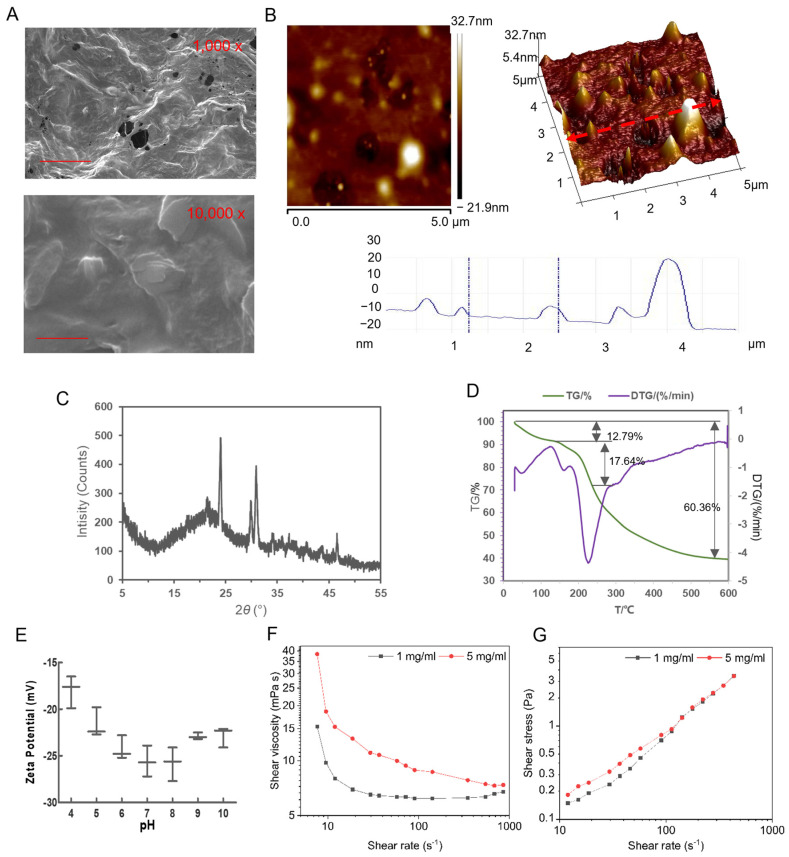
Physicochemical characterization of DIVP. (**A**) SEM images. For top panel, scale bar = 1 μm; for bottom panel, scale bar = 10 μm. (**B**) AFM analysis. Scale bar = 10 μm. (**C**) XRD pattern. (**D**) TG and DTG curves. (**E**) Zeta potential. (**F**,**G**) Rheological properties analysis of DIVP.

SEM images (Figure 4A) reveal a distinct honeycomb-like structure characterized by obvious voids within the polysaccharides, indicating the presence of numerous cavities in DIVP at magnifications of 1000× and 10,000×, respectively. The surface morphology exhibits significant roughness with bulges resembling spheres intertwined with tightly wrapped chains, suggesting a complex structural arrangement for DIVP. The AFM results demonstrate that DIVP adopts a curled (helical) conformation with a spherical appearance and surface cracks (Figure 4B). The chain height ranges from 1.53 to 3.34 nm, while the width varies from 140.16 nm to 278.32 nm. The body diameter fluctuates approximately between 100.64 nm and 317.02 nm with heights ranging from 2.42 nm to 6.21 nm. The three-dimensional image indicates the presence of strong hydrogen bonds between the molecules, which contributed to their aggregation. These findings suggest that DIVP are entangled, forming either large or small rings and branched structures linked by various sugar units. The observed general molecular chain sizes range from 0.1 nm to 1.0 nm [49,50].

XRD patterns can provide valuable insights into the crystal structure of polysaccharides with more pronounced crystal structures yielding stronger diffraction peaks. The XRD pattern of DIVP displays broad diffraction peaks around 21.4° alongside sharp and narrow crystalline diffraction peaks at 24.0°, 29.9°, and 30.9°. The remaining diffraction peaks are relatively flat, indicating that DIVP is a polycrystalline system composed of both crystalline and amorphous structures (Figure 4C). These findings are consistent with the diffraction patterns reported for yeast-derived β-glucan, which exhibited strong diffraction absorption peaks at 6.52°, 19.36°, and 31.89° [51]. Additionally, the presence of a distinct sharp peak at 46.6° suggests a small amount of glyoxalate within the structure [52,53], which is related to its monosaccharide composition. It has been documented that certain regions of polysaccharides exhibit an ordered arrangement during glycosyl polymerization processes, leading to the formation of a crystalline structure. In contrast, other regions lack a clearly ordered arrangement and exhibit an amorphous structure [23].

Thermogravimetric analysis (TGA) is an effective method for evaluating the thermal stability of polysaccharides by monitoring mass changes due to dehydration, decomposition, and oxidation during weight loss at various temperatures. The thermogravimetric (TG) and derivative thermogravimetric (DTG) curves represent, respectively, the ratio of the sample’s mass to its initial mass at the current temperature and the first derivative of points on the TG curve with respect to time. As the temperature increased from 20 °C to 200 °C (Figure 4D), the weight loss in DIVP begin to decrease, ultimately reaching a reduction of 12.79%. At this juncture, a less pronounced decrease in heat loss is observed at 47.3 °C, while a significant increase in heat absorption is noted at 16.8 °C. Subsequently, due to chemical degradation and thermal decomposition processes affecting polysaccharides, including dehydration and depolymerization of the glycan ring, as well as the formation of water and carbon dioxide [52], DIVP experiences approximately 46.65% weight loss between 200 °C and 400 °C. An absorption peak is also observed at 225.6 °C. Following this phase, DIVP is subjected to further heat loss from 401 °C to 600 °C. In this final stage, from 401 °C to 600 °C, the rate of mass loss decreases due to residual polysaccharides involved in additional decomposition processes, leaving a residual mass of approximately 39.64% at 600 °C. These findings suggest that DIVP exhibits excellent thermal stability.

The particle size and zeta potential of polysaccharides are critical indicators for assessing the physical stability of particulate dispersion systems. A higher absolute value of zeta potential indicates greater inter-particle electrostatic repulsion, which correlates with improved physical stability [54]. The average particle size of DIVP is measured at 116.80 nm. Notably, the zeta potential of DIVP surface exhibits fluctuations in response to varying pH levels (Figure 4E). At pH 4, the absolute value of the DIVP zeta potential is approximately 15. This value increases at pH levels above 4, reaching a maximum of 28.2 at pH 8, before gradually decreasing. Even at pH 10, the absolute value remains at 20, indicating that DIVP demonstrates excellent stability in both acidic and alkaline environments.

The viscosity characteristics of the DIVP solution are illustrated in Figure 4F,G. As the concentration of the solution increased, the interactions between the polysaccharide molecules intensified, resulting in a corresponding increase in viscosity. DIVP exhibits a remarkable shear-thinning behavior across a range of shear rates from 0.01 to 20 s^−1^. The apparent viscosity of DIVP solutions decreases as the shear rate increases, which is a hallmark feature of non-Newtonian fluids [53]. This phenomenon can be attributed to disruptions in intermolecular interactions under shear stress conditions and the alignment of polysaccharide molecules along the flow direction. Beyond shear rates exceeding 20 s^−1^, minimal variation in apparent viscosity with increasing shear rate is observed for the DIVP solution, indicating Newtonian behavior [55]. Combined with thermogravimetric analysis and zeta potential assessments, it can be concluded that DIVP demonstrates thermal stability, solution stability, and stability in acid and base environments.

### 3.3. Inhibitory Activity on the Inflammatory Response in Human U937 Macrophages

**Figure 5:**
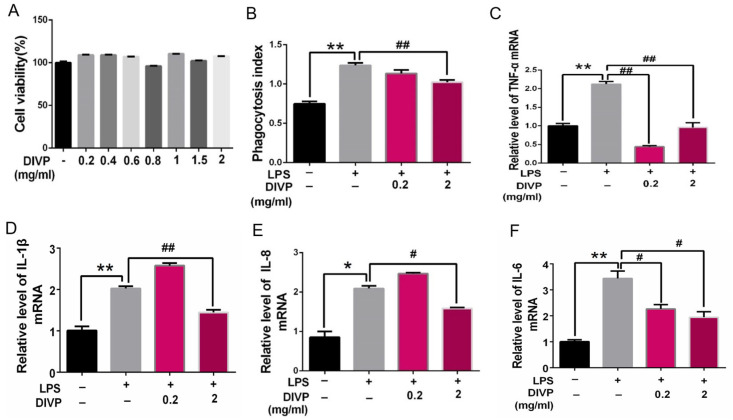
Suppressive effects of DIVP on the inflammatory response in LPS-stimulated U937 cells. (**A**) Cells viability; (**B**) phagocytic activity; (**C**–**F**) the relative mRNA expression level of TNF-α (**C**), IL-1β (**D**), IL-8 (**E**), and IL-6 (**F**). U937 cells were treated with DIVP (0.2 mg/mL or 2 mg/mL) and LPS (10 ng/mL) for 24 h. Light red represents 0.2 mg/mL DIVP and dark red represents 2 mg/mL DIVP; Different color bars represented the different concentrations of DIVP. Statistical significance was determined with a one-way or two-way ANOVA (for dot blot assay), and *p* values are as follows: ns, not significant; * *p* < 0.05, ** *p* < 0.01 vs. control group; ^#^ *p* < 0.05, ^##^ *p* < 0.01 vs. LPS group.

**Figure 6:**
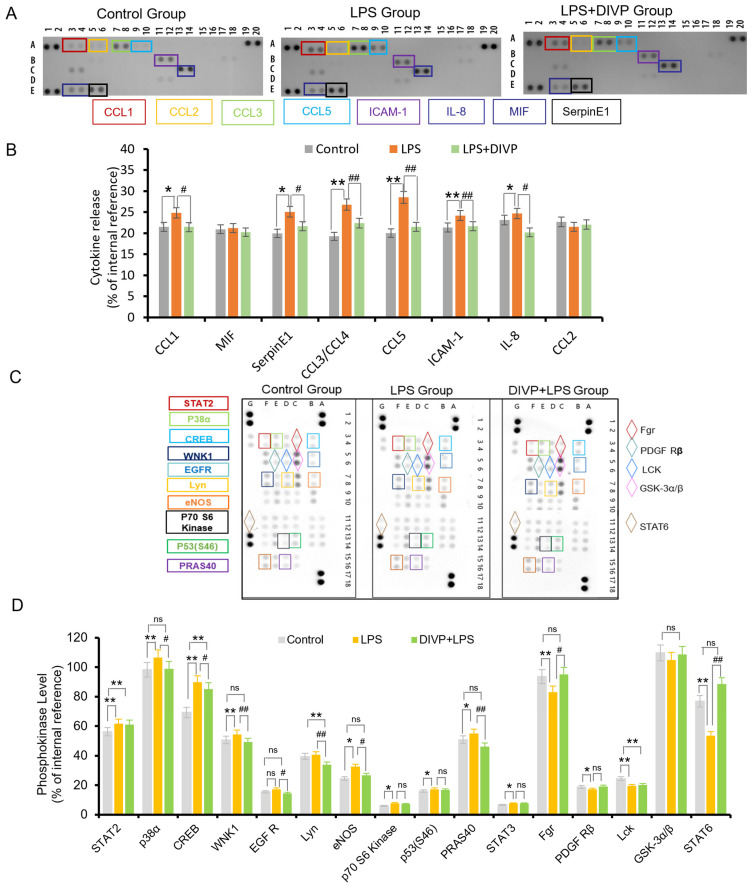
Effects of DIVP on the human cytokines and phosphokinase. (**A**,**B**) Dot blot array analysis (**A**) and quantification (**B**) of pro-inflammatory cytokine secretion from LPS-stimulated U937-macrophages in the presence or absence of 0.2 mg/mL DIVP. (**C**,**D**) Dot blot array analysis (**C**) and relative quantification (**D**) of human phosphokinase from LPS-stimulated U937 macrophages in the presence or absence of 0.2 mg/mL DIVP. A, B, C, D, E in Fig. 6A represent different rows. Statistical significance was determined with a one-way or two-way ANOVA (for dot blot assay), and *p* values are as follows: ns, not significant; * *p* < 0.05, ** *p* < 0.01 vs. control group; ^#^ *p* < 0.05, ^##^ *p* < 0.01 vs. LPS group.

Inflammation, oxidative stress, and dysregulated autophagy are common features associated with NCDs and may serve as key targets for the development of new therapeutic strategies. Macrophages play a crucial role in mediating inflammation and oxidative stress related to NCDs. In this study, PMA-differentiated U937 cells were employed as a cellular model to investigate the effects of DIVP on the inflammatory response. As illustrated in Figure 5A, the control group includes the samples without LPS or DIVP treatment, which are the negative control, and the samples only with LPS treatment are the positive control. DIVP (ranging from 0.2 mg/mL to 2 mg/mL) significantly enhance the activity of U937 cells without inducing any cytotoxic effects. Upon stimulation with LPS, macrophages exhibit a marked increase in their phagocytic ability (Figure 5B). Notably, the combination of DIVP and LPS does not result in excessive activation of U937 cells. The inflammatory response of macrophages can be regulated through various cytokines. The mRNA expression levels of TNF-α, IL-1β, IL-8, and IL-6 are significantly elevated in the LPS-treated group but are dramatically reduced in the DIVP-treated group (*p* < 0.01, Figure 5C–F). DIVP, at a concentration of 0.2 mg/mL, significantly downregulates several LPS-sensitive pro-inflammatory cytokines, including CCL-1, SerpinE1, CCL-3/CCL-4, CCL-5, ICAM-1, and IL-8 (*p* < 0.05, Figure 6A,B). All of these cytokines are involved in both acute and chronic inflammation. Additionally, treatment with DIVP effectively alters the phosphorylation status of 16 out of a total of 45 proteins analyzed (Figure 6C,D). Compared to the normal group, the phosphorylation levels of STAT2, p38α, CREB, WNK1, eNOS, and PRAS40 are significantly increased in the LPS-stimulated groups (*p* < 0.01). However, these phosphorylation levels are markedly lower in the DIVP-treated groups compared to those observed in the LPS-stimulated group (*p* < 0.01, Figure 6C,D). Concurrently, the phosphorylation levels of FGR and STAT6 are significantly inhibited in LPS-stimulated U937 cells, while DIVP is able to reverse this effect. These findings suggest that DIVP can attenuate the inflammatory response by reducing the secretion of inflammatory factors in LPS-induced U937 cells.

The results showed that 1,3-β-glucans were abundant in DIVP, which have exhibited anti-tumor activity [56], anti-bacterial activity [57], anti-oxidant activity [58], and anti-inflammatory activity [59] in the previous studies. For instance, soluble 1,3-β-glucans from *lentinan Grifola frondosa* polysaccharides and Schizophyllum polysaccharides have been widely used in clinical anti-tumor studies and treatment [60]; (1 → 3)-(1 → 6)-β-d-glucans from yeast can alleviate immunosuppression in gemcitabine-treated mice [61]; 1,3-β-glucans from *Trametes versicolor* (L.) *Lloyd* are effective for the prevention of influenza virus infection [62]. In summary, it is reasonable to believe that the polysaccharides in our study also have potential applications in the food and pharmaceutical industries.

## 4. Conclusions

This study elucidated that DIVP consists of Glc, GlcA, Gal, and Man in a molar ratio of 0.535:0.308:0.06:0.047 with a molecular weight of 96.6 kDa. The backbone structure of DIVP is characterized by the repeating unit →3)-β-D-Glc*p*-(1→, featuring branches such as →4)-β-D-Glc*p*-(1→ and →6)-α-D-Gal*p*-(1→, and terminal α-Man*p*-(1→ residues. In addition, DIVP was identified as a semi-crystalline polymer exhibiting excellent physical stability and favorable thermal properties. Notably, DIVP demonstrated moderate anti-inflammatory activity by effectively protecting U937 cells from LPS-stimulated inflammatory responses through downregulating the expression levels of TNF-α, IL-6, IL-8, and IL-1β while regulating the secretion of six chemokines in U937 cells. Additionally, DIVP altered the LPS-induced phosphorylation level of eight proteins.

In conclusion, the peach-shaped *D. indusiata* appears to be a promising source for producing polysaccharides with anti-inflammatory properties. Future research on polysaccharides derived from peach-shaped *D. indusiata* should focus on performing a comprehensive analysis of structure–function relationships as well as the underlying anti-inflammatory effects.

## Figures and Tables

**Table 1:** Primers used in the qRT-PCR analysis.

Target	Forward (5′-3′)	Reverse (5′-3′)
β-actin (human)	ATGTGCAAAAAGCTGGCTTTG	AGATAGCAAATCGGCTGACG
TNF-α (human)	ACGGCATGGATCTCAAAGAC	CGTCAGCCGATTTGCTATCT
IL-1β (human)	ATGGCAACTGTTCCTGAACTCAACT	CAGGACAGGTATAGATTCTTTCCTT
IL-6 (human)	TTCCTCTCTGCAAGAGACT	TGTATCTCTCTGAAGGACT
IL-8 (human)	CTCCAAACCTTTCCACCCC	TCCACAACCCTCTGCACCC

**Table 2:** Linkage types of DIVP obtained by methylation and GC-MS analysis.

	Molar Ratio	RT	Methylated Sugar	Mass Fragments (*m*/*z*)	Type of Linkage
A	0.456	21.426	2,4,6-Me_3_-Glc*p*	45,87, 99, 101, 117, 129, 161, 173, 233	→3)-Glcp-(1→
B	0.112	16.87	2,3,4,6-Me_4_-Glc*p*	45, 71, 87, 101, 117, 129, 145, 161, 205	Glc*p*-(1→
C	0.070	23.578	2,3,4-Me_3_-Gal*p*	45, 87, 99, 101, 117, 129, 161, 189, 233	→6)-Gal*p*-(1→
D	0.056	22.039	2,3,6-Me_3_-Glc*p*	45, 87, 99, 101, 113, 117, 129, 131, 161, 173, 233	→4)-Glc*p*-(1→
E	0.054	26.508	2,3-Me_2_-Glc*p*	45, 71, 85, 87, 99, 101, 117, 127, 159, 161, 201, 261	→4,6)-Glc*p*-(1→
F	0.053	15.311	2,3,4,6-Me4-Man*p*	45, 75, 87, 101, 117, 129, 145, 161, 205	Man*p*-(1→
G	0.051	26.8	2,4-Me_2_-Glc*p*	45, 87, 117, 129, 159, 189, 233	→3,6)-Glc*p*-(1→
H	0.051	28.209	2,4-Me_2_-Gal*p*	45, 87, 117, 129, 159, 189, 233	→3,6)-Gal*p*-(1→
I	0.041	20.751	3,4,6-Me_3_-Glc*p*	45, 71, 87, 99, 101, 129, 145, 161, 189	→2)-Glc*p*-(1→

**Table 3:** Assignments of 1H and 13C NMR chemical shifts of sugar residues in DIVP.

Chemical Shifts (ppm)							
Glycosyl Residues		H1/C1	H2/C2	H3/C3	H4/C4	H5/C5	H6/C6
A	→3)-β-D-Glc*p*-(1→	4.51/95.84	3.12/73.77	3.32/75.23	3.26/77.34	3.56/72.50	3.75/60.44
B	β-D-Glc*p*-(1→	4.39/102.24	3.22/72.61	3.69/70.87	3.39/71.16	3.48/73.52	3.60/60.51
C	→6)-α-D-Gal*p*-(1→	5.09/91.76	3.42/72.98	3.56/80.13	4.07/68.43	3.26/75.70	3.69/60.44
D	→4)-β-D-Glc*p*-(1→	4.86/97.87	3.54/70.43	3.70/73.52	3.31/71.16	3.77/74.79	4.09/68.43
E	→4,6)-β-D-Glc*p*-(1→	4.97/101.58	3.41/72.56	3.64/74.57	3.75/78.06	3.76/73.66	3.64/69.34
F	α-D-Man*p*(1→	5.24/99.32	4.10/74.61	3.52/76.24	3.49/68.73	3.74/76.35	3.86/63.89
G	→3,6)-β-D-Glc*p*-(1→	4.66/101.58	3.34/77.88	3.76/77.52	3.29/70.38	3.41/77.23	4.12/70.61
H	→3,6)-α-D-Gal*p*-(1→	4.99/98.05	3.79/72.79	3.53/79.35	4.01/68.55	3.79/74.81	3.98/71.06
I	→2)-β-D-Glc*p*-(1→	4.73/100.67	3.96/75.16	3.76/79.70	3.43/70.54	3.52/77.14	3.79/62.05

## Data Availability

The original contributions presented in this study are included in the article/Supplementary Materials. Further inquiries can be directed to the corresponding authors.

## Supplementary Materials

The following supporting information can be downloaded at: https://www.mdpi.com/article/10.3390/foods14091536/s1, Figure S1. The flowchart of M&M; Figure S2. MS fragments and inferred residues; Table S1. Raw data used for gene expression analysis.
