# Supplementary material for: Structural Elucidation of Heteropolysaccharides from the Peach-Shaped Dictyophora indusiata and Its Anti-Inflammatory Activity
Source: Foods. 2025 Apr 27;14(9):1536. doi: 10.3390/foods14091536 (PMC12071305; doi:10.3390/foods14091536)
Supplement: Supplementary file 1 [file foods-14-01536-s001.zip › foods-3515041-supplementary.pdf]

Supporting Information

Structural elucidation of a heteropolysaccharides from the peach-shaped Dictyophora indusiata and its anti-inflammatory activity

Ying He[1], Hao Yang[2], Yaxin Liu[1], Yanting Sun[1], Zeguo Feng[1], Xueying Zheng[1], Fei Wang[1], Lei Ma[1], Jianbao Zhang[1], Dan Xu[1], Hui Guo[3], Liguo Qin[2, *], Yali Zhang[1, *]

[a] Key Laboratory of Biomedical Information Engineering of Ministry of Education, Institute of Health and Rehabilitation Science, School of Life Science and Technology, Xi'an Jiaotong University, Xi'an, 710049, China

[b] Key Laboratory of Education Ministry for Modern Design and Rotor-Bearing System, Institute of Design Science and Basic Components, Xi'an Jiaotong University, Xi'an 710049, China

[c] Department of Endocrinology, First Affiliated Hospital of Medical College, Xi'an Jiaotong University, Xi'an 710061, China

[*] Corresponding authors

Yali Zhang, yar.lee@mail.xjtu.edu.cn

LiguoQin, liguoqin@xjtu.edu.cn

Keywords: Peach-shaped phase of *Dictyophora indusiata*; 1,3-β-glucan; human U937 cells; Anti-inflammatory

Figure S1 The flowchart of M&M

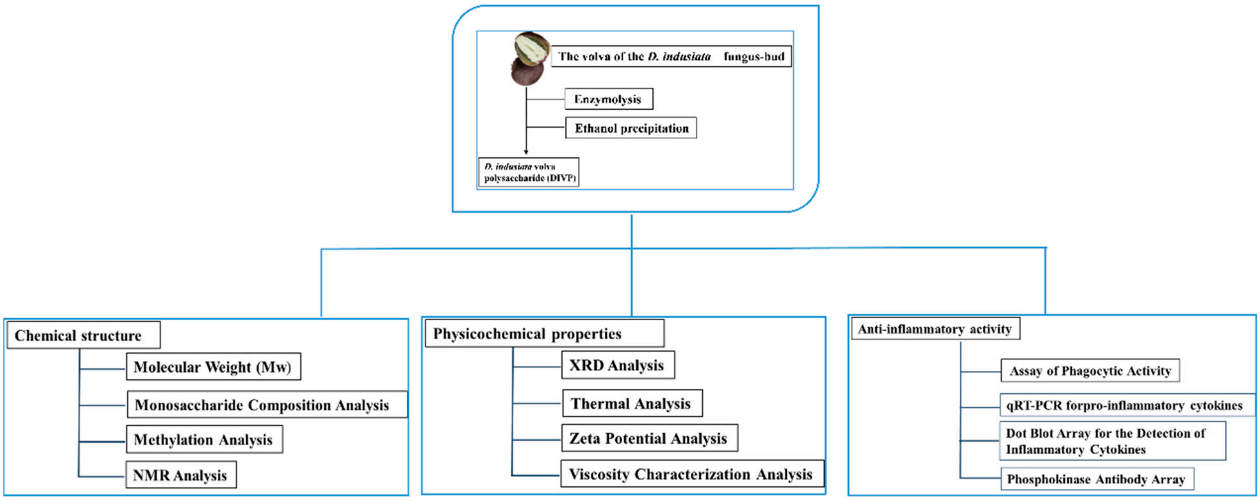

Figure S2

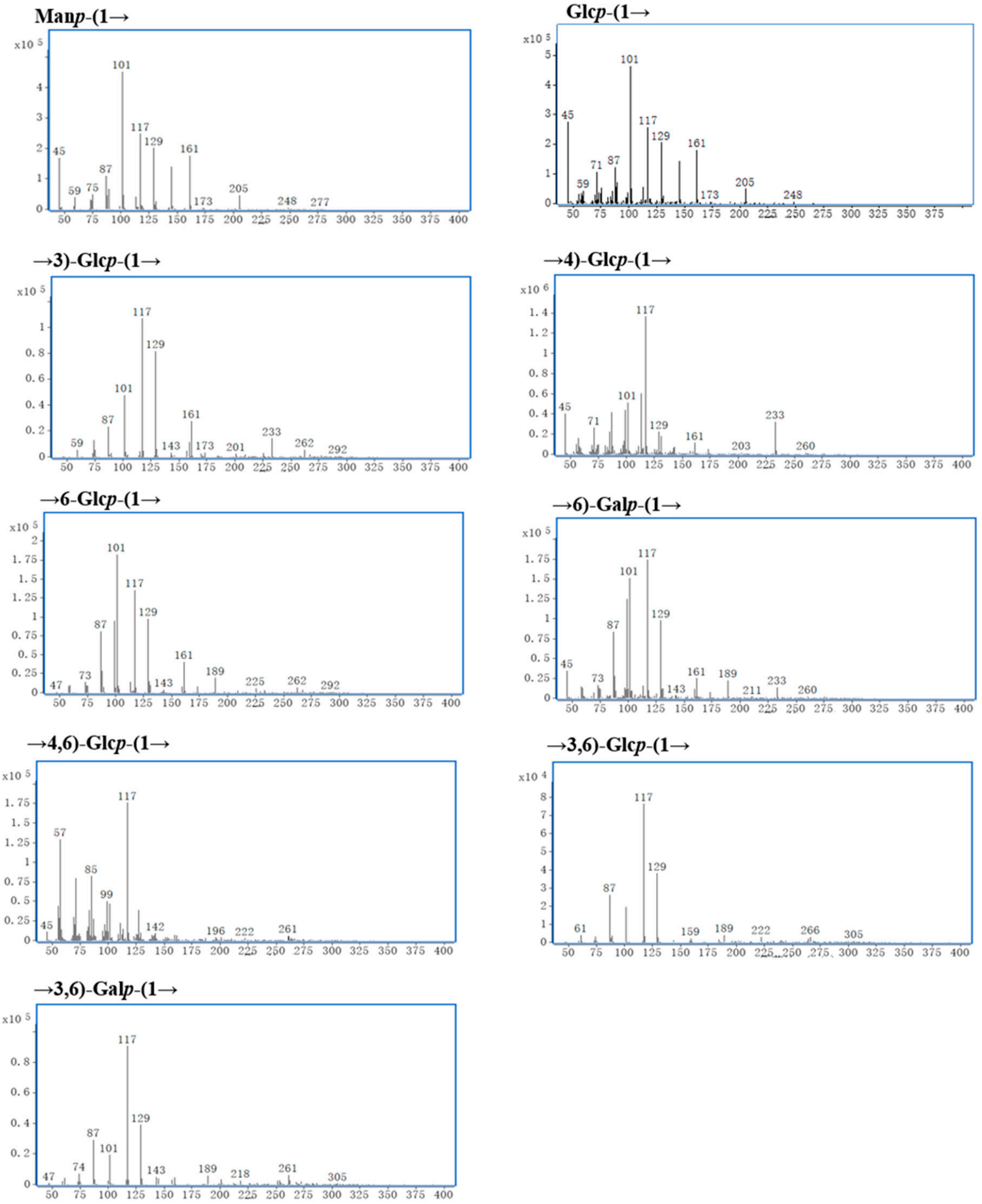

Figure S2 MS fragments and inferred residues

Table S1 Raw data used for gene expression analysis

	β-actin (Cq)	IL-1β (Cq)		β-actin (Cq)	IL-6 (Cq)		β-actin (Cq)	IL-8 (Cq)		β-actin (Cq)	TNF-α (Cq)
control-1	18.87	17.42	control-1	18.87	31.41	control-1	18.87	15.78	control-1	18.87	27.29
control-2	18.80	17.80	control-2	18.80	31.41	control-2	18.80	15.98	control-2	18.80	27.56
control-3	19.01	17.88	control-3	19.01	31.09	control-3	19.01	15.85	control-3	19.01	27.22
LPS-1	18.45	16.14	LPS-1	18.45	29.23	LPS-1	18.45	15.07	LPS-1	18.45	26.78
LPS-2	18.53	16.27	LPS-2	18.53	28.83	LPS-2	18.53	15.11	LPS-2	18.53	26.48
LPS-3	18.28	16.22	LPS-3	18.28	29.10	LPS-3	18.28	15.23	LPS-3	18.28	26.59
0.2mg/ml DIVP+LPS-1	18.44	16.09	0.2mg/ml DIVP+LPS-1	18.65	29.83	0.2mg/ml DIVP+LPS-1	18.44	14.03	0.2mg/ml DIVP+LPS-1	18.44	26.36
0.2mg/ml DIVP+LPS-2	18.95	16.05	0.2mg/ml DIVP+LPS-2	18.83	30.03	0.2mg/ml DIVP+LPS-2	18.95	14.28	0.2mg/ml DIVP+LPS-2	18.95	26.42
0.2mg/ml DIVP+LPS-3	18.58	16.17	0.2mg/ml DIVP+LPS-3	18.87	30.20	0.2mg/ml DIVP+LPS-3	18.58	14.53	0.2mg/ml DIVP+LPS-3	18.58	26.47
2mg/ml DIVP+LPS-1	18.65	16.94	2mg/ml DIVP+LPS-1	18.44	30.28	2mg/ml DIVP+LPS-1	18.65	15.25	2mg/ml DIVP+LPS-1	18.65	27.33
2mg/ml DIVP+LPS-2	18.83	17.13	2mg/ml DIVP+LPS-2	18.95	29.82	2mg/ml DIVP+LPS-2	18.83	15.24	2mg/ml DIVP+LPS-2	18.83	26.92
2mg/ml DIVP+LPS-3	18.87	17.12	2mg/ml DIVP+LPS-3	18.58	30.25	2mg/ml DIVP+LPS-3	18.87	15.28	2mg/ml DIVP+LPS-3	18.87	27.13
